# Systemic and local immune responses in sheep after *Neospora caninum* experimental infection at early, mid and late gestation

**DOI:** 10.1186/s13567-015-0290-0

**Published:** 2016-01-06

**Authors:** David Arranz-Solís, Julio Benavides, Javier Regidor-Cerrillo, Pilar Horcajo, Pablo Castaño, María del Carmen Ferreras, Laura Jiménez-Pelayo, Esther Collantes-Fernández, Ignacio Ferre, Andrew Hemphill, Valentín Pérez, Luis Miguel Ortega-Mora

**Affiliations:** SALUVET, Animal Health Department, Faculty of Veterinary Sciences, Complutense University of Madrid, Ciudad Universitaria s/n, 28040 Madrid, Spain; Livestock Health and Production Institute (ULE-CSIC), 24346 León, Spain; Institute of Parasitology, Vetsuisse Faculty, University of Bern, 3012 Bern, Switzerland

## Abstract

Besides its importance in cattle, *Neospora caninum* may also pose a high risk as abortifacient for small ruminants. We have recently demonstrated that the outcome of experimental infection of pregnant sheep with 10^6^ Nc-Spain7 tachyzoites is strongly dependent on the time of gestation. In the current study, we assessed peripheral and local immune response in those animals. Serological analysis revealed earlier and higher IFN-γ and IgG responses in ewes infected at early (G1) and mid (G2) gestation, when abortion occurred. IL-4 was not detected in sera from any sheep. Inflammatory infiltrates in the placenta mainly consisted of CD8+ and, to a lesser extent, CD4+ T cells and macrophages (CD163+). The infiltrate was more intense in sheep infected at mid-gestation. In the foetal mesenchyme, mostly free tachyzoites were found in animals infected at G1, while those infected in G2 displayed predominantly particulate antigen, and parasitophorous vacuoles were detected in sheep infected at G3. A similar pattern of placental cytokine mRNA expression was found in all groups, displaying a strengthened upregulation of IFN-γ and IL-4 and milder increases of TNF-α and IL-10, reminiscent of a mixed Th1 and Th2 response. IL-12 and IL-6 were only slightly upregulated in G2, and TGF-β was downregulated in G1 and G2, suggestive of limited T regulatory (Treg) cell activity. No significant expression of TLR2 or TLR4 could be detected. In summary, this study confirms the pivotal role of systemic and local immune responses at different times of gestation during *N. caninum* infection in sheep.

**Electronic supplementary material:**

The online version of this article (doi:10.1186/s13567-015-0290-0) contains supplementary material, which is available to authorized users.

## Introduction

*Neospora caninum* is an obligate intracellular protozoan parasite considered as one of the leading infectious causes of abortion in cattle worldwide [1, 2]. Neosporosis is generally asymptomatic in non-pregnant cows; however, the consequences of either primo infection or recrudescence in pregnant cattle may be foetal death or the delivery of a still-born calf or a congenitally infected calf, either healthy or exhibiting nervous clinical signs [3]. It has been agreed that these outcomes depend greatly on the period of gestation in which infection occurs [4]. Several mechanisms have been proposed to lead to foetal death, such as damage directly caused by parasite proliferation in placental and foetal tissues or the immunological imbalance in the placenta [2, 5].

Several reports have shown that a Th1-biased immune response against *N. caninum* is required to control tachyzoite proliferation, involving IFN-γ and IL-12. However, an excess of IFN-γ in the placenta may have detrimental effects for gestation and jeopardise foetal viability [5, 6]. In addition, a Th2-biased cytokine response at the materno-foetal interface may counteract the effects of pro-inflammatory cytokines in order to safeguard foetal viability and hence the maintenance of gestation, yet it may also facilitate parasite proliferation in placental tissues [5, 6]. In addition, the role that the innate immune response plays on intracellular pathogens such as *Neospora* could be sizeable. In fact, activation of *Toll*-*like* receptors (TLR) 2 and 4 leads to the maturation of antigen-presenting cells (APC) and natural killer (NK) cells and pro-inflammatory cytokine production, thus contributing to successful host defence [7, 8]. Nevertheless, relatively little is known in this regard for neosporosis, especially for ovine neosporosis.

On the other hand, although cattle represent the most relevant and economically important target host, recent studies consider *N. caninum* as an important abortifacient also in small ruminants [9], and even the main cause of reproductive losses in some flocks [10, 11]. Moreover, it would be desirable to have a well-established in vivo model for ruminant neosporosis in order to improve the knowledge of the disease, as well as to carry out vaccine or drugs efficacy assays [12]. In this regard, the ovine experimental model of infection provides several advantages over cattle in terms of costs, space, required infrastructure, ease of handling of the animals, the duration of gestation and hence the entire experiment. In a recent study we conducted intravenous experimental infections in pregnant ewes under controlled conditions at three different time points of gestation [13]. The results showed that, in analogy to cattle, the outcome of the infection relied heavily on the time point of gestation that was chosen for infection. Parasitological and pathological findings of these infected ewes and foetuses were also reported [13]. In order to gain further insight into the role that immune responses play in *N. caninum* infected pregnant sheep, our objective in this work was to assess the development of both local and peripheral immune responses after the experimental infections mentioned above.

## Materials and methods

### Experimental design

A full description of the sheep, inocula and experimental design has already been reported in Arranz-Solís et al. [13], which is based on the same animals. Briefly, *Churra* breed ewes seronegative for *N. caninum* and other abortifacient agents were oestrus synchronized and mated with pure breed *Churra* tups for 2 days. Pregnancy and foetal viability were confirmed by ultrasound scanning (US) on day 40 after mating. Pregnant sheep (*n* = 29) were selected and randomly distributed into five experimental groups. Groups 1 (G1 *n* = 6), 2 (G2 *n* = 7) and 3 (G3 *n* = 7) were intravenously (IV) inoculated with 10^6^ culture-derived tachyzoites of the Nc-Spain7 isolate [14] at days 40 (G1), 90 (G2) or 120 (G3) of gestation (dg), respectively. The nine remaining sheep were allocated in groups 4 (G4 *n* = 6) and 5 (G5 *n* = 3) as controls of infection and pregnancy, respectively. Two animals from G4 and one from G5 received an IV inoculum of phosphate-buffered saline (PBS) at each time point of infection. Ewes from G4 were culled at the average time points when abortion took place in their respective group, providing a negative control for further analyses (see below), and ewes from G5 were kept alive until the end of the experiment. Experimental infections were conducted according to the Animal Welfare Committee of the ULE-CSIC, following proceedings described in Spanish and EU legislations at that time (Law 32/2007, R.D. 1201/2005, and Council Directive 2010/63/EU).

### Clinical monitoring and collection of samples

Transabdominal US was used once weekly for the two first weeks post-infection (pi) and then twice weekly to determine foetal viability by monitoring the heartbeat. Blood samples were collected by puncture of the jugular vein in a 10 mL non-heparinised vacutainer tube at days—3, 1, 5, 8, 12, 15, 21 post-infection (pi) and every 2 weeks thereafter until the detection of foetal death or parturition. Serum samples were recovered after centrifugation at 1000 *g* for 10 min and stored at −80 °C for serological analysis. When foetal death was detected, or immediately after parturition, dams and lambs were previously sedated with xylazine (Rompun^®^; Bayer, Mannhein, Germany) and then immediately euthanized by an IV overdose of embutramide and mebezonium iodide (T61^®^; Intervet, Salamanca, Spain). Post-mortem examination of the ewes and lambs was carried out immediately after euthanasia, and foetuses were immediately separated from the placenta. A total of ten randomly selected placentomes were recovered from each placenta and were transversally cut in slices of 2–3 mm of thickness that were distributed for storage in 10% formalin and quick-frozen in cold isopentane for immunohistochemical examinations, and in RNAlater (Sigma–Aldrich, Saint Louis, MO, USA) for cytokine and *Toll*-*like* receptors (TLR) mRNA expression analysis.

### Serological analysis: IgG responses

*Neospora caninum*-specific IgG antibody levels were measured using an in-house indirect enzyme-linked immunosorbent assay (ELISA): soluble antigen prepared according to Álvarez-García et al. [15] was used to coat 96-well microtitre plates. For this, 100 μL/well of antigen at 1.5 μg/mL diluted in carbonate buffer (100 mM, pH 9.6) was incubated overnight at 4 °C. Subsequently, non-specific binding was blocked by adding 300 μL of bovine serum albumin diluted 3% in PBS (pH 7.4) containing 0.05% Tween 20 (PBS-T). After 2 h incubation at room temperature (RT), plates were washed four times with PBS-T. Sera samples were diluted 1:100 in block solution and 100 μL of this dilution was added to each well and incubated for 1 h at 37 °C. In each plate, samples of the same positive and negative control sera were included. After four washes in PBS-T, 100 μL of horseradish peroxidase conjugate protein G (Biorad, Hercules, USA) diluted 1:5000 in PBS-T was added and incubated for 1 h at 37 °C. Plates were washed as above before the addition of 100 μL per well of ABTS substrate (Sigma–Aldrich, Madrid, Spain). The reaction was stopped after 15 min at RT by the addition of 100 μL per well of a solution of 0.3 M oxalic acid, and the optical density (OD) was read at 405 nm (OD_405_). For each plate, values of the OD were converted into a relative index percent (RIPC) using the following formula RIPC = (OD_405_ sample–OD_405_ negative control)/(OD_405_ positive control–OD_405_ negative control) × 100. A RIPC value ≥10 indicates a positive result.

### *N. caninum*-specific IFN-γ and IL-4 responses

IFN-γ and IL-4 levels in sera from dams were measured by the Bovine IFN-γ and IL-4 ELISA development kits (Mabtech AB, Sweden), following manufacturer’s recommendations. Colour reaction was developed by the addition of 3,3′,5,5′-Tetramethylbenzidine substrate (TMB, Sigma–Aldrich, Spain) and incubated for 5–10 min in the dark. Reactions were stopped by adding 2 N H_2_SO_4_. Then, plates were read at 450 nm. The cytokine concentrations were calculated by interpolation from a standard curve generated with recombinant cytokines provided with the kits.

### Immunohistochemistry

For immunohistochemical labeling of parasite antigen, T (CD3 antigen), B (CD79 antigen) and macrophages cells (CD163 antigen), sections were cut from three placentomes per case, infected and control, and placed onto poly-l-Lysine coated slides. Endogenous peroxidase activity was blocked in deparaffinized sections by immersion in 3% hydrogen peroxide in methanol for 30 min in darkness at RT. Rehydrated slides were rinsed twice in PBS. To optimize the immunoreaction, the antigen retrieval was performed using enzymatic or heat-based methods (Additional file 1). After washing the slides twice in PBS, sections were incubated with 100 µL of the primary antibodies diluted in PBS overnight at 4 °C in a humidified chamber. After washing three times in PBS, sections were incubated for 40 min at RT with 100 µL of EnVision^®^+/HRP solution (Dako North America Inc, Carpinteria, USA). After washing twice in PBS, antibody localization was determined using 100 µL of 3,3-diaminobenzidine (DAB, Sigma–Aldrich Corp.) as chromogenic substrate for peroxidase. Sections were counterstained with haematoxylin for 30 s and mounted.

For the characterization of T cell subpopulations of the inflammatory infiltrate found in infected sheep, CD4 and CD8 antigens were immunolabelled on cryostat sections from frozen samples. Steps followed for the immunohistochemical labeling were the same as those for paraffin sections except deparaffination and antigen retrieval, which are not necessary in cryostat sections. As a number of ewes showed detached placentas at the time of necropsy that showed a degree of autolysis [13], those three placentomes that preserved the best histological architecture within each infected group (G1, G2 and G3) and sections of three placentomes from each control ewe were selected for CD4 and CD8 characterization.

Lymphocyte (T and B cells and also CD4+ and CD8+ populations) and macrophage quantification was performed under a light microscope with final magnification of 400× in the three placentomes examined per animal. The number of labelled cells was counted in ten random fields within the endometrial chorioallantoic interdigitation area of the placentome. The amount of *N. caninum* antigen was subjectively evaluated in the same placentomes per animal, paying special attention to its distribution in the different structures of the placentome (maternal vs foetal areas of the placenta).

### RNA extraction and reverse transcription

Total RNA was extracted from approximately 10 mg of five placentomes using the commercial Maxwell^®^ 16 LEV simplyRNA Purification Kit, developed for automated Maxwell^®^ 16 System (Promega, Wisconsin, USA), following the manufacturer’s recommendations. It included a DNAse treatment step to avoid genomic DNA contamination of the RNA samples. For all samples, RNA concentrations were determined by spectrophotometry (Nanophotometer, Implen), and the integrity of the RNA was checked by the 260/280 absorbance ratio (close to 2.0) and the visualization of the 18S and 28S ribosomal subunits after electrophoresis on a 1% agarose gel.

Reverse transcription was carried out by the master mix SuperScript^®^ VILO™ cDNA Synthesis Kit (Invitrogen, Paisley, UK) in a 20 μL reaction using up to 2.5 μg of total RNA. Obtained cDNA reactions were diluted 1:10 and used in quantitative PCR (qPCR) assays.

### Quantitative real-time PCR (qPCR)

In order to analyse cytokine and TLR mRNA expression, primers for ovine IFN-γ, IL-4, IL-10, IL-12p40, TNF-α, IL-6 and TGF-β1 cytokines, the *Toll*-*like* receptors (TLR) 2 and 4, and the housekeeping gene β-actin were used (Additional file 2). Primer3Plus software [16] was used to design primers, which were checked with chromosomal and mRNA sequences using the BioEdit Sequence Alignment Editor v.7.1.3 (Tom Hall, Ibis Therapeutics, Carlsbad, CA, USA). For all target genes, except for TLR2 and TLR4, at least one primer annealed at intron splice junctions or at largely separated exons (for IL-12p40) to prevent amplifications of genomic DNA. For TLR2 and TLR4, to ensure that cDNA samples were not contaminated with genomic DNA, reactions were set up using 500 ng of non-reverse transcribed RNA instead of cDNA. Failure to generate a detectable signal indicated the sample to be DNA free. Real-time PCR reactions were performed in 25 μL using 12.5 μL of the Power SYBR^®^ Green PCR Master Mix (Applied Biosystems, Foster City¸ CA, USA), 10 pmol of each primer and 5 μL of diluted cDNA samples (1/10) in an ABI 7300 Real Time PCR System (Applied Biosystems), with the following amplification conditions: 95 °C for 10 min followed by 40 cycles at 95 °C for 15 s and 60 °C for 1 min. In the case of TLR2 and TLR4, the methodology previously described by Menzies and Ingham [17] was followed. For each target gene, a seven-point standard curve was included in each batch of amplifications based on tenfold serial dilutions starting at 10 ng/µL of plasmid DNA in which the full-length cDNA containing the gene fragments used as templates in qPCR were cloned. Average Ct values were used to determine mRNA expression for each sample. For all groups, after checking RNA integrity and measuring β-actin mRNA levels (housekeeping), the three best placentomes were selected for each ewe and analysed for the rest of cytokines and TLRs. The cytokine mRNA expression level was calculated by the interpolation of the average Ct in plasmid standard curves and then adjusted to the number of fg per ng of total RNA/cDNA equivalent. The relative quantification of cytokine mRNA expression levels (x-fold change in expression) was carried out by the comparative 2^−ΔΔCt^ method, as described previously [18].

### Statistical analysis

In order to compare the analysis of local and peripheral immune response in infected ewes, data from both control groups (G4 and G5) were merged into one group. Antibody responses for each experimental group were analysed using the one-way ANOVA test. When statistically significant differences were found, a Tukey’s Multiple Range test was applied to examine all possible pairwise comparisons at each sampling time. Variations in IFN-γ and IL-4 levels from sera were analysed by the repeated measures two-way ANOVA test until day 15 pi. Cytokine and TLR mRNA expression levels as well as the presence of each cell population in placenta were analysed using the non-parametric Kruskal–Wallis test, followed by a Dunn’s multiple range test for all pairwise comparisons. In addition, to assess differences between each group with the control group, a Dunn’s multiple comparison test was performed for each cytokine or TLR. Finally, additional differences between CD4+ and CD8+ considering all infected ewes together (G1 + G2 + G3) were assessed by a Mann–Whitney test.

Statistical significance for all analysis was established with *P* < 0.05. All statistical analyses were carried out using GraphPad Prism 6.01 software (San Diego, CA, USA).

## Results

A summary of the clinical course, lesional development and parasite distribution already reported in Arranz-Solís et al. [13] is shown in Additional file 3.

### Specific anti-*Neospora* IgG antibody responses

**Figure 1:**
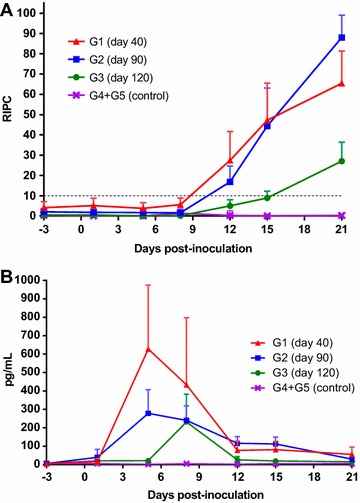
**Humoral and cellular immune responses in sera.** Graphs representing **A** IgG and **B** IFN-γ levels in sera from ewes inoculated with 10^6^ Nc-Spain7 tachyzoites at day 40 (G1), 90 (G2) and 120 (G3) of gestation, as well as control groups (G4 + G5). Each point represents the mean + SD at the different sampling times for each group. Data beyond 21 dpi is not represented since several animals did not maintain pregnancy and were therefore sacrificed. Sera levels of total IgG antibodies against *N. caninum* are expressed as a relative index percent (RIPC), according to the formula: RIPC = (OD_405_ sample–OD_405_ negative control)/(OD_405_ positive control–OD_405_ negative control) × 100. Concentrations of IFN-γ are expressed in pg/mL.

The results on *N. caninum* specific IgG antibody responses are shown in Figure 1A. IgG levels increased significantly from day 12 pi in G1 (*P* < 0.001) and G2 (*P* < 0.05) compared to control groups (G4 + G5), and continued rising until day 21 pi, whereas in G3, significant IgG levels were only detected from day 21 pi (*P* < 0.01). IgG levels remained high in all groups when foetal death/birth took place (data not shown). Comparison between infected groups showed significantly lower average values of IgG in ewes in G3 compared to G1 at days 12, 15 and 21 pi (*P* < 0.001), and compared to G2 at days 15 and 21 pi (*P* < 0.001). In addition, significant differences were also found between G1 and G2 on day 21 pi (*P* < 0.01), with increased IgG levels in the latter group. All control animals (G4 and G5) showed basal levels throughout the experimental study.

### IFN-γ and IL-4 kinetics in sera

A significant increase of IFN-γ levels in sera was observed on days 5 and 8 pi for G1 and G2 (*P* < 0.001), and on day 8 pi for G3 (*P* < 0.01) compared to control groups (G4 + G5). These levels decreased to basal values in all three infected groups from day 12 pi and maintained low levels onwards (Figure 1B). Kinetics within each group also showed that maximum IFN-γ levels were reached earlier in G1 and G2 (5 dpi) than in G3 (8 dpi). In addition, average levels of IFN-γ were significantly higher in G1 compared to both G2 and G3 at days 5 (*P* < 0.001) and 8 (*P* < 0.01) pi, and in G2 compared to G3 at day 5 pi (*P* < 0.001). Regarding IL-4 responses, there were no changes in levels detected for any infected group (G1–G3) in comparison to basal levels (prior to infection and control animals) (data not shown). None of the control animals (G4 and G5) showed IFN-γ or IL-4 levels above basal levels recorded prior to inoculation throughout the experimental study.

### Characterization of inflammatory cell populations in placenta

#### T cells (CD3+, CD4+, CD8+)

**Figure 2:**
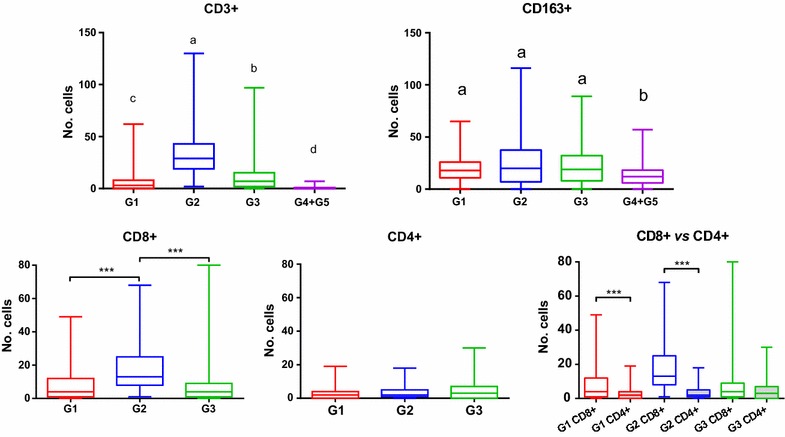
**Quantification of immunohistochemically labelled T lymphocytes and macrophages in the placenta.** Box-plot graphs representing the median number of cells, the lower and upper quartiles (boxes) and minimum and maximum values (whiskers) of CD3+, CD163+, CD8+ and CD4+ (see legends) cell populations in placentas after intravenous infection of ewes with 10^6^ Nc-Spain7 *N. caninum* tachyzoites at day 40 (G1), 90 (G2) and 120 (G3) of gestation. Uninfected control groups (G4 + G5) are also represented. Different letters (a to d) above each column indicate significant differences among groups (*P* < 0.01). (***) indicates *P* < 0.001 significant differences between groups.

**Figure 3:**
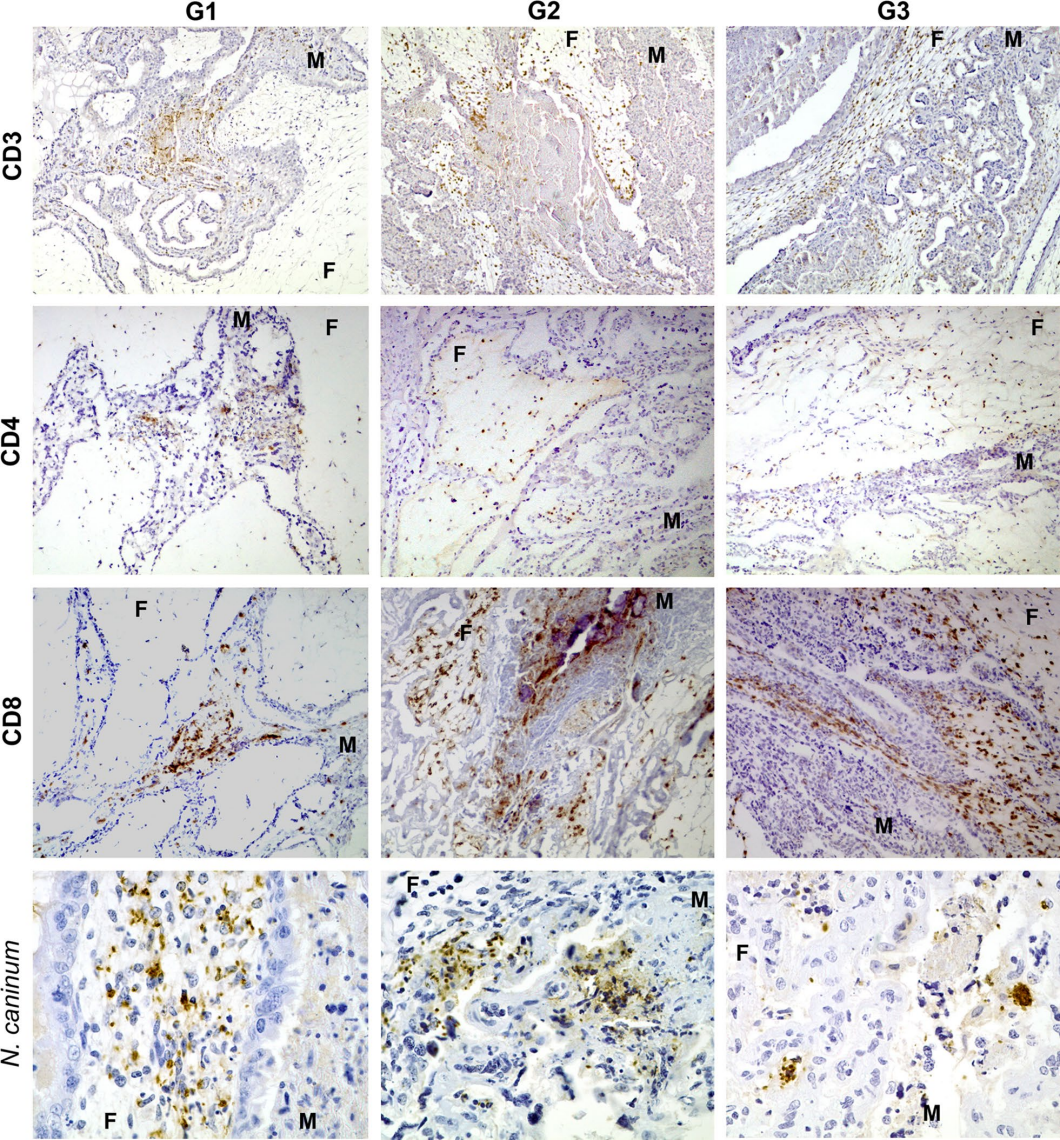
**Comparison of the immunohistochemical labelling of T inflammatory cells and parasite antigen in the placenta.** This panel compares the distribution and frequency of T lymphocytes (CD3, CD4 and CD8 antigens) and parasite antigen immunohistochemically labelled in samples from the three groups. Pictures showing the labelling of T cells (first three rows) were taken at 75× and those of the parasite antigen (last row) were taken at 150×. F: foetal mesenchyme, M: maternal endometrial stalk.

CD3+ lymphocytes were found in all the placentas examined from animals in the three periods of gestation. Their number was significantly higher in those infected than those from the non-infected controls (*P* < 0.001), where they were scant. The infiltrate of T cells was more evident in the placentas from G2, where the count of positive cells was significantly higher than in G1 (*P* < 0.001) and G3 (*P* < 0.01) (Figure 2). There were also differences in the distribution of the positive cells. In placentomes from G1, the group with a lower number of positive cells, these were found mainly in the maternal placenta at the endometrial stalk between the crypts, as a multifocal infiltrate in relation to lesions (focal areas of necrosis and infiltration of inflammatory cells). On the other hand, in G2 and G3, CD3+ cells were found to be abundant in the mesenchyme of the foetal villi as aggregates, but also as a diffuse infiltrate in both the foetal mesenchyme and, to a lesser extent, in the connective tissue of the maternal caruncle (Figure 3). CD3+ cells were more frequent in the vicinity of lesions, occasionally forming aggregates, but, mostly, they did not infiltrate the necrotic foci.

Regarding T cell subpopulations in infected groups, CD8+ cells were significantly more abundant in G2 than in the other two groups (*P* < 0.001), while there were no significant differences regarding CD4+ cells (Figure 2). In G1, both populations of T lymphocytes appeared associated to lesions in the maternal placenta, while in G2 and G3 these lymphocytes were found mainly in the foetal mesenchyme. In these two groups, CD8+ T lymphocytes were found mainly as a diffuse infiltrate of scattered cells but also, occasionally, as multifocal aggregates related to lesions (Figure 3). A similar distribution was observed for CD4+ cells, but they were fewer and more dispersed (Figure 3). At large, the number of CD8+ cells was higher than CD4+ cells when all infected animals were compared together (*P* < 0.001). Moreover, individual comparison among groups revealed a higher number of CD8+ than CD4+ in all infected groups, although these differences were only statistically significant for G1 and G2 (*P* < 0.001) (Figure 2). CD4+ and CD8+ labelling in control animals showed very few cells randomly distributed in the maternal stalks of the placentome, mainly in relation to vessels. There were so few cells that only one or two random fields showed positive cells. Subjective evaluation of the entire slide suggested that the number of CD8+ cells was higher, although no statistical analysis was possible due to the scarcity of random fields with positively labelled cells.

#### Macrophages (CD163+)

Cells labelled for CD163 were found in the placentas of both infected and non-infected control animals at the three periods of gestation. While inoculated animals showed positive cells in both the interdigitate tissues and endometrial areas of the placentome, in the non-infected sheep CD163+ cells were mainly found at the base of the placentome in the maternal endometrium, where they were scattered. Inoculated animals showed a higher number of macrophages than non-infected (*P* < 0.001), although no significant differences were observed between infected groups (Figure 2). In the placentomes from G1, positive cells where found in the foetal mesenchyme, in relation to parasites, and in the caruncular septae, where they were distributed around vessels. In G2 and G3 the distribution of macrophages was similar, being more numerous in the former, but not showing statistically significant differences (Figure 2). They were scattered in the foetal mesenchyme and also forming aggregates associated to necrotic lesions in the foetal or maternal villi, while in the caruncular septa they were located around vessels.

#### B cells (CD79αcy)

CD79+ cells were very rare in all the placentas studied and no differences were observed between animals, either infected or non-infected.

### Parasite antigen distribution

Immunohistochemical labelling of *Neospora* antigen in placentomes from G1 revealed multifocal distribution of the parasite in the foetal villi. Each of these foci (3–6 per placentome examined) was formed by numerous 5-to-10 μm wide parasitophorous vacuoles or tachyzoites-like structures localized in the mesenchyme (Figure 3). In the areas where these parasite structures were found, there was also an increase in the number of inflammatory cells, mainly in the adjacent maternal stalks but also, to a lesser extent, in the close foetal mesenchyme where the parasite was found. This infiltrate was mainly formed by T lymphocytes (CD3+) and macrophages (CD163+). In the trophoblast layer adjacent or close to these areas, the amount of parasite-like structures was lower. In G2, there were clearly less parasite-like structures than in G1. Instead, particulate antigen (amorphous granular debris positively labelled) in relation to necrotic lesions was the main finding (Figure 3). Finally, in G3, parasite-like structures were labelled in the areas adjacent to the lesion, in a similar amount than in G2. There was no particulate antigen in this group (Figure 3).

### Cytokine and TLR mRNA expression levels in placental tissues

To characterize the immune response at the foeto-maternal interface after *Neospora* infection in all thirds of gestation, we measured mRNA expression in placentomes of IFN-γ, IL-4, IL-10, IL-12p40, TNF-α, IL-6 and TGF-β1 cytokines, as well as TLR2 and TLR4. The selected placentomes from both infected and uninfected ewes showed no differences regarding β-actin mRNA expression (Additional file 4). Those RNA samples with a significant decrease in the β-actin gene expression, suggestive of poor RNA quality by autolysis, were removed from the analysis (1 ewe of G1, 2 of G2 and 2 of G3).

**Figure 4:**
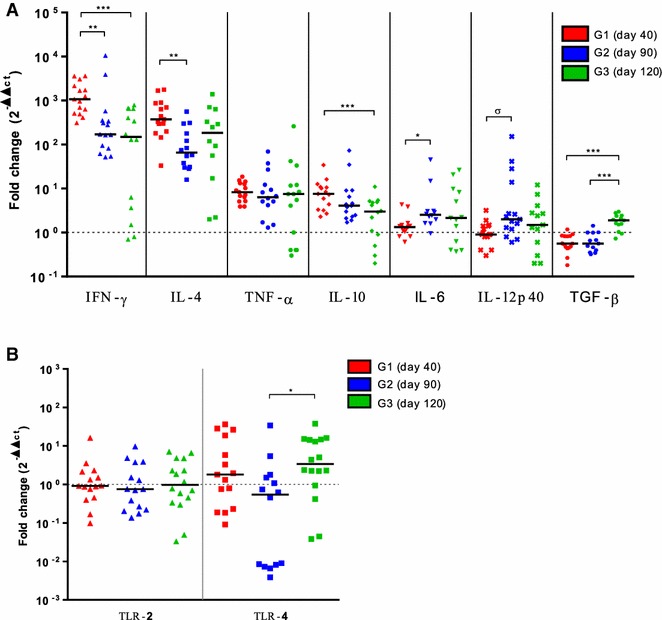
**Placental cytokine and** ***Toll*** **-** ***like*** **receptor transcript expression.** Scatter-plot graphs of relative mRNA expression levels (as x-fold change) of **A** IFN-γ, IL-4, TNF-α, IL-10, IL-6, IL12p40 and TGF-β1 cytokines and **(B)** *Toll*-*like* receptors (TLR) 2 and 4 in placenta after intravenous infection of ewes with 10^6^ Nc-Spain7 *N. caninum* tachyzoites at day 40 (G1), day 90 (G2) and day 120 (G3) of gestation. Data are represented as individual points. Horizontal lines represent median values for each group. The horizontal discontinuous line set at 10^0^ indicates uninfected animals baseline. (***), (**), (*) and (σ) symbols indicate *P* < 0.001, *P* < 0.01, *P* < 0.05 significant differences and a tendency to significance (*P* = 0.052), respectively.

In terms of fold-change (i.e., 2^−ΔΔCt^), a significant increase in the expression levels of all cytokines in the three infected groups was observed, except for IL-12 and IL-6 in G1 and IL-10, IL-12 and TGF-β in G3, which showed no significant differences with uninfected animals. By contrast, TGF-β expression levels in G1 and G2 were significantly downregulated (*P* < 0.01) (Figure 4A). Regarding TLR2 and TLR4 expression levels, no significant differences were observed when comparing infected and non-infected groups (Figure 4B).

When comparing cytokine expression levels within each group, a similar pattern was observed. The most highly increased cytokines were IFN-γ (by 311–3622 fold for G1, 51.7–10460 for G2 and 0.7–783 for G3) and IL-4 (by 33–1764 fold for G1, 15.9–562 for G2 and 2–1393 for G3), followed by modest increases in TNF-α (by 3.9–18.7 fold in G1, 1.3–69.5 for G2 and 0.3–259 for G3). Remarkably, while the IFN-γ/IL-4 ratio at the materno-foetal interface in G1 and G2 showed an evident IFN-γ-biased response, this ratio was more balanced in G3, being significantly lower compared to both G1 (*P* < 0.01) and G2 (*P* < 0.001) (Additional file 5).

In order to study the influence of the period of gestation on the cytokine expression at the materno-foetal interface, further comparison of the cytokine mRNA levels revealed that G1 exhibited significantly higher levels of IFN-γ than G2 (*P* < 0.01) and G3 (*P* < 0.001), higher IL-4 levels than G2 (*P* < 0.01), and increased IL-10 levels than G3 (*P* < 0.05) (Figure 4A). By contrast, IL-6 expression levels were significantly higher in G2 compared to G1 (*P* < 0.05), while TGF-β were significantly lower in G1 and G2 compared to G3 (*P* < 0.001). Finally, no differences were found in the expression levels of IL-12 and TNF-α between infected groups, except a tendency to significance between G1 and G2 for IL-12 (*P* = 0.052) (Figure 4A). Similarly, no differences were detected in TLR2 and TLR4 mRNA levels other than slightly higher values of TLR4 in G3 compared to G2 (*P* < 0.05) (Figure 4B).

## Discussion

We have recently shown that the outcome of experimental infection in pregnant ewes was highly dependent on the timing of infection during gestation [13]. To gain further insight into ovine neosporosis, our objective in the present study was to study the immune response induced by the experimental infections in the first (G1), second (G2) and third (G3) terms of gestation. As expected, our results supports the hypothesis that the immune responses play a pivotal role in the possible outcome scenarios of *N. caninum* infection during pregnancy.

The analysis of the peripheral immune response showed an early IFN-γ production in all dams infected early in gestation (G1), which decreased to non-detectable levels from the second week pi, coinciding with a gradual increase of specific IgG levels. These results demonstrate a clear predominance of IFN-γ levels during the innate immune response. Similar kinetics have been described in previous reports carried out in cattle infected with *Neospora*, where an early increase of IFN-γ production, with a noticeable predominance over IL-4 production, was detected upon stimulation of peripheral blood mononuclear cells (PBMC) between days 5 and 7 pi [19–23]. The lack of detection of peripheral IL-4 in our study may be explained by the low, and thus undetectable, amounts present in sera, although it could also be due to different responses between sheep and cattle. On the other hand, and similarly to reports on both cattle and sheep infections [8, 23, 24], IgG levels increased from the second week pi onwards until foetal death took place (19–21 dpi).

The analysis of placentas from ewes infected with *N. caninum* at early gestation in our previous work had demonstrated the highest parasite burdens but only mild lesions, which might be attributed to the short time frame between infection and abortion [13]. Moreover, IHC analysis confirmed the high amount of tachyzoite-like structures, mainly as free tachyzoites, and showed that the parasite was mostly located in the foetal part of the placenta. Likewise, in a recent study carried out in cattle infected with the same isolate Nc-Spain7, parasite burden was found to be significantly higher in the cotyledon [23]. This fact can be accounted for the still immature foetal immune system at this stage, which may allow the parasite to cross the placental barrier before the maternal immune response is established, and freely multiply in foetal tissues without restriction [13]. Indeed, the lowest amount of inflammatory cells was found in this group (G1) and, interestingly, these were located almost exclusively in the maternal part of the placentome. It seems clear that the immune response elicited in the placenta somewhat controlled parasite multiplication in the maternal area, but not in the foetal one, as observed by IHC. Altogether, these facts point to the parasite uncontrolled multiplication in foetal tissues, and probably the damage caused by this, as the cause of abortion in ewes infected at early gestation [13]. On the other hand, severe lesions and diffuse infiltration of inflammatory cells in the placenta have been described in cattle upon infection at early gestation [19, 21, 23, 25]. This fact may indicate that, in addition to foetal tissue damage, there could be also a local immune-mediated component in the placenta participating in the pathogenesis of ovine abortion, since very few and only mild lesions were observed in ovine placentas, contrarily to bovine placentas.

In this respect, the cytokine transcript expression profile at the materno-foetal interface in ewes infected at early gestation showed a strong upregulation of IFN-γ and IL-4 mRNA, and milder increases of TNF-α and IL-10, confirming that a mixed Th1- and Th2-type immune response took place. By contrast, no significant expression of IL-6 and IL-12 transcripts could be detected. Comparison with similar studies carried out in cattle, since none have been done in sheep, showed a similar upregulation of IFN-γ, IL-4, TNF-α and IL-10, but also an increase in IL-12 expression levels [23, 26, 27]. Although several parameters other than host species could also be implicated, it is conceivable that IL-12 may have a less important role for the placental immune response in sheep compared to cattle, or, most probably, that its expression may rapidly decrease following an early peak of expression just after infection. Furthermore, the highest IFN-γ mRNA levels at the materno-foetal interface were found in ewes from G1. This fact has been already reported in studies comparing cattle infected at days 70 and 210 of gestation [26, 28]. As argued above, the high expression levels of IFN-γ transcripts could be due to the high parasite burden found in placentas of ewes from this group, which efficiently stimulates a Th1-biased immune response. In addition, the uncontrolled tachyzoite proliferation in the foetal part of the placentome could have been continuously stimulating the immune response in the maternal area. On the other hand, the expression of regulatory cytokine transcripts in dams from G1 displayed a contrasting behaviour, with IL-10 being upregulated and TGF-β downregulated. The increment of IL-10 has been previously described in bovine infections, suggesting that it could be secreted as a response to the high IFN-γ levels, and, to a lesser extent, TNF-α Th1-type cytokines [23, 26, 27, 29]. Similarly, a relation between parasite burden and IL-10 induction in placenta has been postulated in the pregnant mouse model, as a means of regulation of the Th1 immune response [30]. By contrast, TGF-β is considered a signature cytokine for the activity of regulatory T cells, acting as a growth inhibitor [31]. Therefore, this downregulation may be consequence of low suppressive functions of Tregs at the materno-foetal interface, which can potentially facilitate an IFN-γ biased Th1/Th2 balance with detrimental effects on pregnancy [6, 32].

Regarding the infection at mid gestation (G2), similar kinetics in the peripheral immune responses to G1 were observed. The *N. caninum*-specific IgG antibody responses were maintained high until foetal death occurred (34–48 dpi). Nevertheless, IFN-γ production showed lower levels compared to ewes from G1, indicating a weaker initial immune response stimulation in G2. This fact might have led to a lower initial control of parasitaemia at the peripheral level, allowing a higher number of parasites to reach the placenta [6, 33]. This, in turn, may have resulted in a higher initial stimulation of the immune response at the materno-foetal interface in an attempt to control parasite dissemination to the foetus. As we pointed out in our previous work, the more severe lesions together with the lower parasite burden found in placentas from G2 suggests that the inflammatory response developed in this organ has been effective in controlling tachyzoite proliferation, maybe because of the longer time frame between infection and abortion, but it has also damaged placental tissues [13]. In accordance to this, IHC analysis showed a scarce labelling of parasite antigen, mainly found as particulate antigen instead of intact tachyzoites. This particulate antigen most likely originated from tachyzoites that were degraded by immune effectors. Moreover, placentas from G2 harboured the highest numbers of inflammatory cells such as T lymphocytes. In addition, the development of the foetal immune system at this stage could also have aided the maternal immune response to partially control the transplacental transmission, yet not to a protective degree. This correlates with the parasite burden and lesions found in foetal organs from this group, especially in the liver, which showed to be much lower compared to those from G1 [13]. Taken together, our results suggest that in ewes infected at mid-gestation (G2) foetal death was most likely a consequence of the severe lesions elicited in the placenta.

On the other hand, one striking finding of this study was the predominance of CD8+ over CD4+ cells. Previous studies in cattle and buffaloes found the opposite [27, 34–36]. Moreover, CD4+ cells have been shown to be more relevant in the protection against neosporosis [37]. The reason for the high number of CD8+ cells in sheep is not clear. It has been found that CD8+ cells could predominate over CD4+ as early responders to *N. caninum* (48 h pi) [38]. However, in the present study the infection was not in an early stage. Why CD8+ cells are more abundant than CD4+ in the ovine placenta when abortion due to neosporosis occurs deserves further investigations.

The cytokine profile observed in placentas of ewes infected at mid gestation showed to be very similar to that of G1, yet, by and large, it was less intense in G2. The only difference in this regard was the slight upregulation of IL-6 and IL-12 found in this group, which was not observed neither in G1 nor G3. Previous studies in bovine neosporosis and murine toxoplasmosis also revealed an upregulation of IL-6 in placentas at mid-gestation [30, 39]. On the other hand, previous studies in cattle have described much higher increases of IL-12 in dams after *N. caninum* infection. However, the predominance of a Th1-biased response, described to be the most efficacious to control intracellular parasites such as *Neospora*, was apparently ineffective to control the parasite proliferation in placentas of G1 and G2. Nevertheless, in contrast to G1, ewes from G2 displayed lower cytokine expression levels, which, in addition to the upregulation of IL-6, may be a consequence of the partial control of the parasite achieved at the materno-foetal interface.

As for infection at late gestation (G3), the IgG response in ewes was less pronounced and was elicited at a later time point compared to G1 and G2. These differences may suggest that at late gestation there was a different regulation of the immune response elicited against the parasite, which could be accounted for by the mature foetal immune system and the advanced gestational stage. Likewise, the IFN-γ response was slightly delayed (day 7 pi), but was at a similar level as the G2 IFN-γ response. On the other hand, parasite burden and lesions found in placenta from this group, yet lower and less frequent, were similar to those observed in G2 [13]. However, IHC demonstrated that only few parasites were associated to lesions and, similarly, only few inflammatory cells were found, mainly located in the foetal part of the placenta. These differences in comparison to G2 may be explained by the higher capacity of the foetus to control the parasite at this stage, but also to the shorter time window between infection and delivery of lambs. Therefore, it is tempting to hypothesise that the triggering of birth avoided the recovery of foetuses from infection in this group, as implied by the premature delivery of the three weak lambs that exhibited higher parasite burdens and lesions compared to the other six lambs of the group [13].

Under this scenario, the immune response at the materno-foetal interface revealed a different pattern when compared to the other groups with respect to the IFN-γ/IL-4 ratio, IL-10 and TGF-β. The IFN-γ/IL-4 ratio, biased towards IFN-γ in G1 and G2 (when foetal death occurred), was reduced in the last term (G3), as both cytokines were transcribed at similar levels. This fact has not been described in cattle, where a similar IFN-γ/IL-4 mRNA ratio, biased towards IFN-γ, is present in the placenta after *N. caninum* infection at both early and late gestation [26]. The balance between Th1 and Th2 cytokine expression has been suggested to be of paramount importance in determining the severity and outcome of the disease following *N. caninum* infection [21, 40]. Since no other reports in this regard are available in sheep, further studies are needed to elucidate the importance of the IFN-γ/IL-4 ratio in the control of the parasite and its detrimental effects to the foetus. On the other hand, the decreased IL-10 levels, in addition to the fact that TGF-β mRNA levels remained stable (in contrast to G1 and G2), may be suggestive of a diminished regulatory function in the placenta, which is in accordance to the balanced IFN-γ/IL-4. Notably, this more balanced ratio in sheep from G3 did not limit lesions, or only to a little extent, observed in placenta, but avoided the occurrence of abortion [13]. Finally, the analysis of TLR2 and TLR4 mRNA in the placenta showed no significant expression in any infected sheep. These receptors recognize bacteria and protozoa [41], and have been suggested to play an important role in innate immune response in human placenta [42]. Furthermore, an increase in TLR2 expression has been found in maternal lymph nodes 56 days after *N. caninum* infection in cattle at late gestation [8]. The lack of expression in the ovine experimental model could suggest that these receptors do not possess an important role in the recognition of *N. caninum* in the placenta or in sheep, or more likely, that their activation takes place at a different time frame.

In conclusion, this is the first work in sheep comparing the immune responses that are elicited after *N. caninum* infection during the three trimesters of gestation under the same standardized conditions. Our results suggest that while abortion in G1 may be mainly caused by the uncontrolled parasite multiplication in the foetus, abortion in G2 is more likely to be triggered by the severe lesions developed in the placenta. The fact that no abortions occurred in G3 could be due to the short time span between infection and birth of lambs and the more mature foetal immune system. In addition, very similar antibody responses and cytokine and cell population profiles have been previously described in cattle. However, a number of differences between sheep and cattle are evident, such as the much lower IL-12 secretion and the inverted balance of the CD4+/CD8+ population ratio in ovine placentas. These results shed new light into the pathogenesis of ruminant neosporosis and pose interesting questions that certainly warrant further research.

## Additional files

10.1186/s13567-015-0290-0 **Primary antibodies used in the immunohistochemical labelling of antigens in the placentas.** ^1^ Bio-Rad Laboratories, Inc. USA; ^2^ Dako Cytomation, Glostrup, Denmark; ^3^ AbD Serotec, Oxford, UK; ^a^ Heat Induced Epitope Retrieval; ^b^ Cryostat sections; ^c^ Trypsin incubation.
10.1186/s13567-015-0290-0 **Sequences of primers used for cytokine real-time PCR (qPCR) and standard curve data.** ^a^ NCBI accession numbers are for ovine cDNA sequences used in primer design. Primer annealing was also checked with the *Ovis aries* genomic DNA sequences of the chromosome 2 for TLR4, the chromosome 3 for IFN-γ, the chromosome 4 for IL-6, the chromosome 5 for IL-4 and IL12p40, the chromosome 12 for IL-10, the chromosome 14 for TGF-β1, the chromosome 17 for TLR2, the chromosome 20 for TNF-α and the chromosomes 14 and 24 for β-actin in NCBI database [43]. ^b^ Minimal coefficient of regression (*R* ^*2*^) of standard curves for each PCR target in all batches of amplification, based on tenfold dilutions (10^−1^–10^−7^) of 10 ng/µL from plasmid stocks. Ct values increased linearly until the level of 10^−7^ dilution of all plasmids. ^c^ Standard curve slopes. Minimal and maximal values for slopes for each PCR target in all batches of amplification. ^d^ Inter-assay coefficient of variation. CV values indicate the maximum and minimum CVs of all points from standard curves for each PCR target run in this study. Subscript numbers indicate curve point for CV values. (*) Indicates primers annealing at intron splice junctions. No amplification products were detected when ovine genomic RNA free-DNA samples were tested with cytokine primers (data not shown). ^1, 2, 3^ Primer first described by Regidor-Cerrillo et al. [23], Rosbottom et al. [26] and Menzies and Ingham [17], respectively. No number superscript indicates primers designed in this study.
10.1186/s13567-015-0290-0 **Summary of lesion and PCR detection and quantification of** ***N. caninum*** **in placenta and foetal liver and brain**. dpi: days post infection when abortion occurred; ASF: Average size of focus; %LES: Percentage of section affected by lesions. * All lambs from this group gave birth to viable lambs between days 142 and 155 of gestation. Three of nine lambs were born prematurely prior to day 145, and exhibited weakness, recumbency and unresponsiveness to external stimuli. ^1^ Percentage of the section affected by lesions. ^2^ Fractions represent the number of positive animals/total number of animals checked by nested-ITS1 PCR, and figures within brackets represent the median values of parasite burden (tachyzoites/mg tissue). ^a, b^ Values followed by unlike superscripts differ significantly by Dunn’s test for pairwise comparisons. ^c, d^ Fractions determined for positive animals followed by unlike superscripts differ significantly by Fisher’s exact test.
10.1186/s13567-015-0290-0 **Housekeeping gen β-actin mRNA expression levels in placentomes.** Box-plot graph of the β-actin mRNA expression levels in placentomes from infected (G1, G2 and G3) and uninfected (G4 and G5) ewes, expressed as femtograms (fg). No significant differences were observed between groups.
10.1186/s13567-015-0290-0 **Placental IFN-γ/IL-4 ratio.** IFN-γ/IL-4 ratio in placentas after intravenous infection of ewes with 10^6^ Nc-Spain7 *N. caninum* tachyzoites at day 40 (G1), day 90 (G2) and day 120 (G3) of gestation. Data are represented as individual points. One data point from G2 is outside axis limits (ratio of 35.8) and is therefore not shown on the figure. Horizontal lines represent median values for each group. (***) and (**) symbols indicate *P* < 0.001 and *P* < 0.01 significant differences, respectively.
