# Supplementary material for: Systemic and local immune responses in sheep after Neospora caninum experimental infection at early, mid and late gestation
Source: Vet Res. 2016 Jan 6;47:2. doi: 10.1186/s13567-015-0290-0 (PMC4702303; doi:10.1186/s13567-015-0290-0)
Supplement: Supplementary file 1 — 10.1186/s13567-015-0290-0 Primary antibodies used in the immunohistochemical labelling of antigens in the placentas. 1 Bio-Rad Laboratories, Inc. USA; 2 Dako Cytomation, Glostrup, Denmark; 3 AbD Serotec, Oxford, UK; a Heat Induced Epitope Retrieval; b Cryostat sections; c Trypsin incubation. [file 13567_2015_290_MOESM1_ESM.docx]

| Antigen | Target | mAb information | Dilution |
| --- | --- | --- | --- |
| CD163 | Monocytes/macrophages | MCA1853^1, a^ | 1:200 |
| CD3 | Total T lymphocytes | Polyclonal^2, a^ | 1:200 |
| CD4 | CD4 T lymphocytes | CC30^3, b^ | 1:250 |
| CD8 | CD8 T lymphocytes | CC38.65^3, b^ | 1:500 |
| CD79_αcy_ | Total B cells | HM57^2, a^ | 1:25 |
| *N. caninum* | Parasite | In house^c^ | 1:3000 |

**Additional file 1 antibodies used in the immunohistochemical labelling of antigens in the placentas.**

^1^ Bio-Rad Laboratories, Inc. USA

^2^ Dako Cytomation, Glostrup, Denmark

^3^ AbD Serotec, Oxford, UK

^a^ Heat Induced Epitope Retrieval

^b^ Cryostat sections

^c^ Trypsin incubation
