# Supplementary material for: Systemic and local immune responses in sheep after Neospora caninum experimental infection at early, mid and late gestation
Source: Vet Res. 2016 Jan 6;47:2. doi: 10.1186/s13567-015-0290-0 (PMC4702303; doi:10.1186/s13567-015-0290-0)
Supplement: Supplementary file 2 — 10.1186/s13567-015-0290-0 Sequences of primers used for cytokine real-time PCR (qPCR) and standard curve data. a NCBI accession numbers are for ovine cDNA sequences used in primer design. Primer annealing was also checked with the Ovis aries genomic DNA sequences of the chromosome 2 for TLR4, the chromosome 3 for IFN-γ, the chromosome 4 for IL-6, the chromosome 5 for IL-4 and IL12p40, the chromosome 12 for IL-10, the chromosome 14 for TGF-β1, the chromosome 17 for TLR2, the chromosome 20 for TNF-α and the chromosomes 14 and 24 for β-actin in NCBI database [43]. b Minimal coefficient of regression (R 2) of standard curves for each PCR target in all batches of amplification, based on tenfold dilutions (10−1–10−7) of 10 ng/µL from plasmid stocks. Ct values increased linearly until the level of 10−7 dilution of all plasmids. c Standard curve slopes. Minimal and maximal values for slopes for each PCR target in all batches of amplification. d Inter-assay coefficient of variation. CV values indicate the maximum and minimum CVs of all points from standard curves for each PCR target run in this study. Subscript numbers indicate curve point for CV values. (*) Indicates primers annealing at intron splice junctions. No amplification products were detected when ovine genomic RNA free-DNA samples were tested with cytokine primers (data not shown). 1, 2, 3 Primer first described by Regidor-Cerrillo et al. [23], Rosbottom et al. [26] and Menzies and Ingham [17], respectively. No number superscript indicates primers designed in this study. [file 13567_2015_290_MOESM2_ESM.docx]

| **Target^a^** | **Primer** | **Primer sequences (5’-3’)** | **Product size (bp)** | ***R*^2 b^** | **Slope^c^** | **CV (%)^d^** |
| --- | --- | --- | --- | --- | --- | --- |
| **IFN-γ** (X52640.1) | QIFN-UP*^,1^ | GATTCAAATTCCGGTGGATG | 110 | 0.996 | (-3.40) – (-3.20) | 2.26_(-6)_ – 1.68_(-2)_ |
|  | QIFN-RP^1^ | TTCTCTTCCGCTTTCTGAGG |  |  |  |  |
| **IL-4** (XM_004008636.1) | QIL4ov-UP* | CTGCCCCAAAGAACGCAACT | 154 | 0.996 | (-3.62) – (-3.48) | 7.24_(-1)_ - 0.95_(-6)_ |
|  | QIL4-RP*^,2^ | TCATTCACAGAACAGGTCTTGCTT |  |  |  |  |
| **IL-10** (NM_001009327.1) | QIL10-UP*^,2^ | TGCTGGATGACTTTAAGGGTTACC | 60 | 0.997 | (-3.23) – (-3.22) | 7.22_(-1)_ – 2.33_(-7)_ |
|  | QIL10-RP^1^ | AAAACTGGATCATTTCCGACAAG |  |  |  |  |
| **IL-12p40** (NM_001009438.1) | QIL12-UP^2^ | ATGGAATTTGGTCCACTGATATT | 95 | 0.997 | (-3.33) – (-3.20) | 4.22_(-2)_ – 1.10_(-5)_ |
|  | QIL12-RP^2^ | GTGAAGTGTCCAGAATAATCCTTT |  |  |  |  |
| **TNF-α** (NM_001024860.1) | QTNF-UP*^,1^ | CCAGAGGGAAGAGCAGTCC | 126 | 0.996 | (-3.24) – (-3.19) | 6.69_(-2)_ – 2.28_(-7)_ |
|  | QTNFov-RP* | GGAGCGCTGATGTTGGCTAC |  |  |  |  |
| **IL-6** (X68723.1) | QIL-6-UP* | CTGGGTTCAATCAGGCGATT | 150 | 0.998 | (-3.38) – (-3.33) | 3.31_(-1)_ - 0.04_(-4)_ |
|  | QIL-6-RP | GGATCTGGATCAGTGTTCTGA |  |  |  |  |
| **TGF-β1** (NM_001009400.1) | QTGF-UP* | GGTGGAATACGGCAACAAAA | 117 | 0.998 | (-3.47) – (-3.36) | 2.06_(-1)_ - 0.35_(-6)_ |
|  | QTGF-RP | CGAGAGAGCAACACAGGTTC |  |  |  |  |
| **TLR-2** (NM_001048231.1) | QTLR2-UP^,3^ | ACGACGCCTTTGTGTCCTAC | 192 | 0.997 | (-3.74) – (-3.47) | 2.95_(-6)_ – 0.84_(-2)_ |
|  | QTLR2-RP^3^ | CCGAAAGCACAAAGATGGTT |  |  |  |  |
| **TLR-4** (NM_001135930.1) | QTLR4-UP^3^ | ACTGACGGGAAACCCTATCC | 208 | 0.997 | (-3.41) – (3.35) | 1.03_(-6)_ – 0.75_(-3)_ |
|  | QTLR4-RP^3^ | CAGGTTGGGAAGGTCAGAAA |  |  |  |  |
| **β- actin** (NM_001009784.1) | BACTIN-UP*^,1^ | ACACCGCAACCAGTTCGCCAT | 216 | 0,992 | (-3.63) – (-3.46) | 2.23_(-1)_-0.20_(-6)_ |
|  | QBACT216-RP^1^ | GTCAGGATGCCTCTCTTGCT |  |  |  |  |

**Additional file 2 Sequences of primers used for cytokine real-time PCR (qPCR) and standard curve data.**

^a^ NCBI accession numbers are for ovine cDNA sequences used in primer design. Primer annealing was also checked with the *Ovis aries* genomic DNA sequences of the chromosome 2 for TLR4, the chromosome 3 for IFN-γ, the chromosome 4 for IL-6, the chromosome 5 for IL-4 and IL12p40, the chromosome 12 for IL-10, the chromosome 14 for TGF-β1, the chromosome 17 for TLR2, the chromosome 20 for TNF-α and the chromosomes 14 and 24 for β-actin in NCBI database [43].

^b^ Minimal coefficient of regression (*R^2^*) of standard curves for each PCR target in all batches of amplification, based on 10-fold dilutions (10^−1^−10^−7^) of 10 ng/µL from plasmid stocks. Ct values increased linearly until the level of 10^-7^ dilution of all plasmids.

^c^ Standard curve slopes. Minimal and maximal values for slopes for each PCR target in all batches of amplification.

^d^ Inter-assay coefficient of variation. CV values indicate the maximum and minimum CVs of all points from standard curves for each PCR target run in this study. Subscript numbers indicate curve point for CV values.

(*) Indicates primers annealing at intron splice junctions. No amplification products were detected when ovine genomic RNA free-DNA samples were tested with cytokine primers (data not shown).

^1, 2, 3^ Primer first described by Regidor-Cerrillo et al. [23], Rosbottom et al. [26] and Menzies and Ingham [17], respectively. No number superscript indicates primers designed in this study.
