# Supplementary material for: Systemic and local immune responses in sheep after Neospora caninum experimental infection at early, mid and late gestation
Source: Vet Res. 2016 Jan 6;47:2. doi: 10.1186/s13567-015-0290-0 (PMC4702303; doi:10.1186/s13567-015-0290-0)
Supplement: Supplementary file 3 — 10.1186/s13567-015-0290-0 Summary of lesion and PCR detection and quantification of N. caninum in placenta and foetal liver and brain. dpi: days post infection when abortion occurred; ASF: Average size of focus; %LES: Percentage of section affected by lesions. * All lambs from this group gave birth to viable lambs between days 142 and 155 of gestation. Three of nine lambs were born prematurely prior to day 145, and exhibited weakness, recumbency and unresponsiveness to external stimuli. 1 Percentage of the section affected by lesions. 2 Fractions represent the number of positive animals/total number of animals checked by nested-ITS1 PCR, and figures within brackets represent the median values of parasite burden (tachyzoites/mg tissue). a, b Values followed by unlike superscripts differ significantly by Dunn’s test for pairwise comparisons. c, d Fractions determined for positive animals followed by unlike superscripts differ significantly by Fisher’s exact test. [file 13567_2015_290_MOESM3_ESM.docx]

**Additional file 3 Summary of lesion and PCR detection and quantification of *N. caninum* in placenta and foetal liver and brain**.

| Group | Foetal death (dpi) | **Placenta** | |  | **Liver** | |  | **Brain** | |
| --- | --- | --- | --- | --- | --- | --- | --- | --- | --- |
|  |  | %LES^1^ | PCR^2^ |  | %LES^1^ | PCR^2^ |  | %LES^1^ | PCR^2^ |
| G1 (day 40) | 19-21 | 0.25%^b^ | 6/6 (7888^a^) |  | 3.37%^a^ | 11/11^c^ (6318^a^) |  | 0.05% | 11/11 (1697^a^) |
| G2 (day 90) | 34-48 | 9.07%^a^ | 7/7 (308.5^b^) |  | 0.02%^b^ | 5/12^d^ (0.01^b^) |  | 0.18% | 10/12 (0.25^b^) |
| G3 (day 120) | born* | 5.74%^a^ | 7/7 (190.8^b^) |  | 0.16%^b^ | 4/9^d^ (0.01^b^) |  | 0.25% | 6/9 (0.01^b^) |

dpi: days post infection when abortion occurred; ASF: Average size of focus; %LES: Percentage of section affected by lesions.

* All lambs from this group gave birth to viable lambs between days 142 and 155 of gestation. Three of nine lambs were born prematurely prior to day 145, and exhibited weakness, recumbency and unresponsiveness to external stimuli.

^1^ Percentage of the section affected by lesions.

^2^ Fractions represent the number of positive animals/total number of animals checked by nested-ITS1 PCR, and figures within brackets represent the median values of parasite burden (tachyzoites/mg tissue).

^a, b^ Values followed by unlike superscripts differ significantly by Dunn’s test for pairwise comparisons.

^c, d^ Fractions determined for positive animals followed by unlike superscripts differ significantly by Fisher’s exact test.
