# Supplementary figures and images for: Systemic and local immune responses in sheep after Neospora caninum experimental infection at early, mid and late gestation
Source: Vet Res. 2016 Jan 6;47:2. doi: 10.1186/s13567-015-0290-0 (PMC4702303; doi:10.1186/s13567-015-0290-0)

## Slide 1
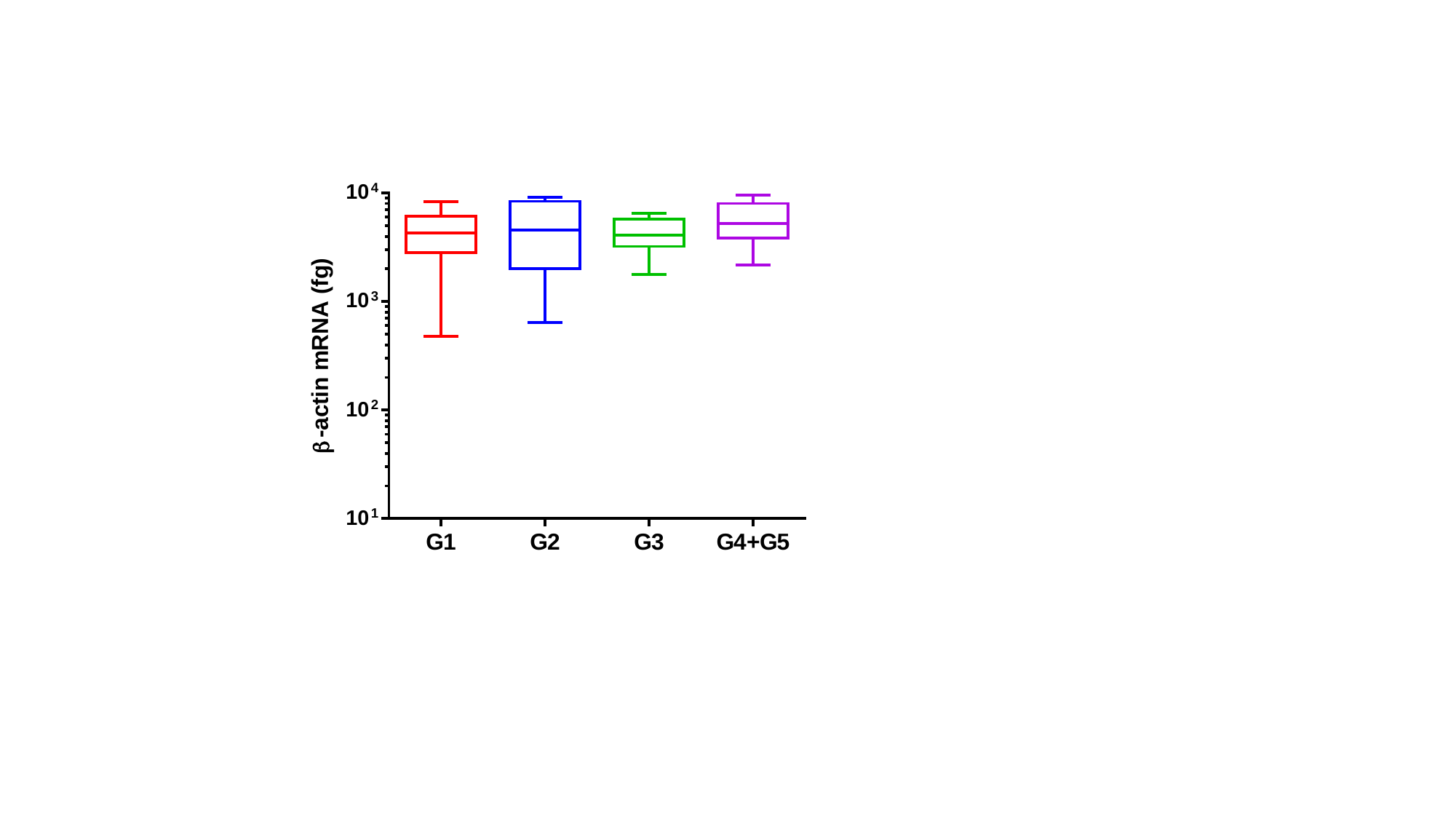

Supplement: Supplementary file 4 — 10.1186/s13567-015-0290-0 Housekeeping gen β-actin mRNA expression levels in placentomes. Box-plot graph of the β-actin mRNA expression levels in placentomes from infected (G1, G2 and G3) and uninfected (G4 and G5) ewes, expressed as femtograms (fg). No significant differences were observed between groups. [file 13567_2015_290_MOESM4_ESM.pptx]

## Slide 1
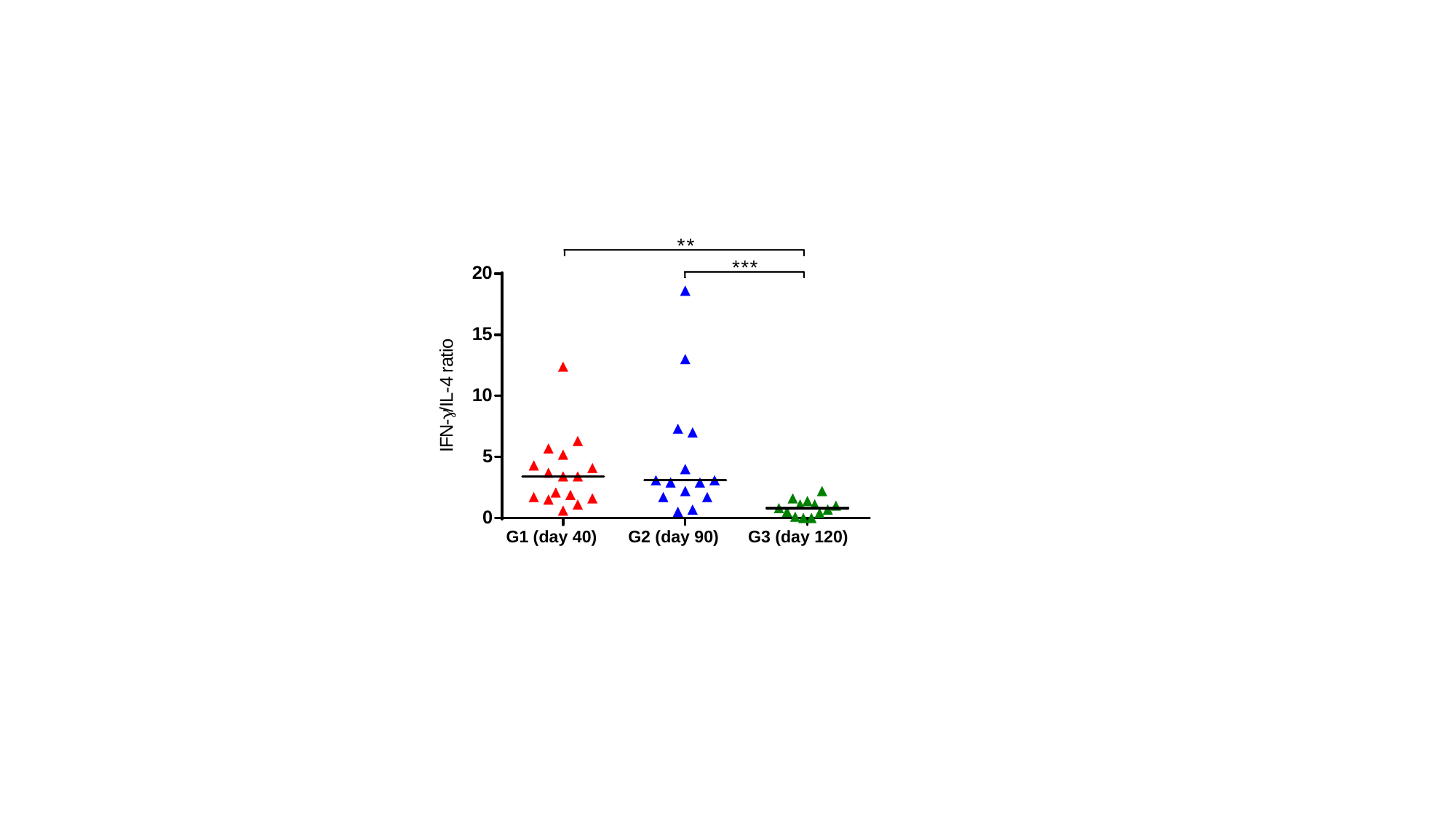

Supplement: Supplementary file 5 — 10.1186/s13567-015-0290-0 Placental IFN-γ/IL-4 ratio. IFN-γ/IL-4 ratio in placentas after intravenous infection of ewes with 106 Nc-Spain7 N. caninum tachyzoites at day 40 (G1), day 90 (G2) and day 120 (G3) of gestation. Data are represented as individual points. One data point from G2 is outside axis limits (ratio of 35.8) and is therefore not shown on the figure. Horizontal lines represent median values for each group. (***) and (**) symbols indicate P < 0.001 and P < 0.01 significant differences, respectively. [file 13567_2015_290_MOESM5_ESM.pptx]
